# Beta-agonist overuse and delay in obtaining medical review in high risk asthma: a secondary analysis of data from a randomised controlled trial

**DOI:** 10.1038/s41533-017-0032-z

**Published:** 2017-05-11

**Authors:** Janine Pilcher, Mitesh Patel, Alison Pritchard, Darmiga Thayabaran, Stefan Ebmeier, Dominick Shaw, Peter Black, Irene Braithwaite, Mark Weatherall, Richard Beasley

**Affiliations:** 1 Medical Research Institute of New Zealand, Wellington, New Zealand; 2 Capital & Coast District Health Board, Wellington, New Zealand; 3 Nottingham Respiratory Research Unit, School of Medicine, University of Nottingham, Nottingham, UK; 4 Chest Clinic, Derriford Hospital, Plymouth, UK; 5 University of Auckland, Auckland, New Zealand; 6 University of Otago, Wellington, New Zealand

## Abstract

Asthma mortality surveys report delays in seeking medical review and overuse of beta-agonist therapy as factors contributing to a fatal outcome. However, the strength of these associations is limited because many asthma deaths are unwitnessed. We undertook a secondary analysis of data from a 24-week randomised controlled trial of 303 patients with high-risk asthma, randomised to combination budesonide/formoterol inhaler according to a single maintenance and reliever therapy regimen or fixed dose budesonide/formoterol with salbutamol as reliever (Standard) regimen. Medication use was measured by electronic monitors. The thresholds for high, marked and extreme beta-agonist use days were defined in the single maintenance and reliever therapy arm as: >8, >12 and >16 actuations of budesonide/formoterol in excess of four maintenance doses, respectively; and in the Standard arm as: >16, >24 and >32 actuations of salbutamol, respectively. Whether a medical review was obtained within 48 h of an overuse episode was determined by review of data collected during the study by participant report. The mean (standard deviation) proportion of days in which high, marked and extreme beta-agonist overuse occurred without medical review within 48 h was 0·94(0·20), 0·94(0·15) and 0·94(0·17), and 0·92(0·19), 0·90(0·26) and 0·94(0·15) for single maintenance and reliever therapy and Standard regimens, respectively. In at least 90% of days, in which beta-agonist overuse occurred, patients did not obtain medical review within 48 h of beta-agonist overuse, regardless of the magnitude of overuse or the inhaled corticosteroid/long-acting beta-agonist regimen.

## Reliever inhaler overuse and delay in medical review in asthma

In asthma, overuse of beta-agonist reliever medication and delay in seeking medical review in an exacerbation are linked to asthma deaths. Janine Pilcher at the Medical Research Institute of New Zealand, and co-workers, conducted a review of data from a study of 303 adult patients with severe asthma, followed over 24 weeks. The patients were allocated to either a budesonide/formoterol, or a salbutamol inhaler to take for symptom relief, in addition to their maintenance treatment. Inhalers were fitted with electronic monitors, to accurately document every use. In both groups, on 90% of days when an exacerbation requiring excess use of an inhaler occurred, patients did not follow-up with medical professionals within 48 h as advised. Further, in both groups, ‘extreme’ reliever inhaler use was recorded at least once in around one in four patients.

## Introduction

Asthma mortality surveys report that delay in seeking medical review is a common and important factor contributing to a fatal outcome.^1–5^ Beta-agonist overuse immediately before asthma death has also been reported, but the strength of this association is limited because these surveys rely on reports from relatives and friends of patients who have died from asthma.^1–5^ Furthermore, many asthma deaths are unwitnessed. Prescription database surveys identify a strong association between increased dispensing of short-acting beta-agonist drugs and the risk of mortality.^6–10^ The risk of asthma death increases markedly when more than 1.4 inhaler canisters per month are dispensed.^7^ Although this is estimated to be equivalent to an average of ten actuations per day, this may not correspond to actual patterns of use during a severe exacerbation. Patients with asthma also often report taking very high doses of beta-agonists during severe exacerbations leading to hospital admission, but these estimates may be influenced by recall bias.^11^ In the situation of stable asthma, patients who overuse their medications are likely to under-report actual use and conversely those who underuse tend to over-report actual use.^12^

The best method to assess actual patient self-administration of beta-agonist is to use validated electronic monitors that record the exact date and time of each actuation.^13, 14^ This approach has been used in one 13-week observational study in children^15^ and also in one 24-week randomised controlled trial (RCT) of two regimens based on maintenance combination budesonide/formoterol inhaler therapy in high risk adult asthma.^16^ In this RCT, we reported long-term patterns of beta-agonist use^16^ as well as use during severe exacerbations leading to hospital attendance.^17^ We identified that in less than 10% of days when high beta-agonist use occurred, defined as > 16 actuations of salbutamol (or equivalent) in a 24-h period, did patients obtain medical review within 48 h, despite this advice being verbally explained and documented in the asthma action plans provided.^16^ About one in four patients self-administered >32 actuations per day of salbutamol (or equivalent) on at least one occasion during the study. These findings showed the extent of unsupervised beta-agonist overuse by patients at risk of severe asthma exacerbations. In a subsequent analysis, we identified that very high doses of beta-agonist were commonly self-administered by patients without seeking medical review within 2 weeks before hospital presentation with a severe exacerbation of asthma.^17^

The objectives of this current analysis were to investigate the incidence of delay in obtaining medical review at different levels of excessive beta-agonist use throughout the 24 weeks of the RCT, and to describe patterns of beta-agonist use before and after severe exacerbations requiring systemic corticosteroid therapy. Our hypothesis was that most episodes of beta-agonist use, in excess of the thresholds at which medical review was recommended in the implemented asthma action plans, would not result in patients obtaining medical care.

## Results

The participants in the RCT are described in Table 1. There were 151 participants randomised to the single maintenance and reliever therapy (SMART) regimen and 152 participants to the Standard regimen.

**Table 1:** Participant characteristics

Characteristic	SMART group (*N* = 151)	Standard group (*N* = 152)
	Mean [(standard deviation (SD)]	
Age (years)	41·3 (13·7)	42·6 (14·5)
ACQ-5 Score	1·78 (0·99)	1·79 (2·4)
Daily ICS dose (budesonide or equivalent), µg	805 (353)	813 (370)
On-treatment FEV_1_ (L)	2·62 (0·91)	2·50 (0·78)
On-treatment FEV_1_ % predicted	81·6 (18·9)	80·4 (20·5)
Severe exacerbations in the prior 12 months	1·55 (1·31)	1·73 (1·22)
	Median (IQR)	
Baseline self-reported reliever use^a^	2 (1 to 3)	2 (1 to 3)
Number of hospital admissions ever for asthma	1 (0 to 4)	1 (0 to 4)
	N/151 (%)	N/152 (%)
Male	48 (31·8)	46 (30·3)
Ethnicity		
European	113 (74·8)	118 (77·6)
Māori	25 (16·6)	19 (12·5)
Pacific Islander	5 (3·3)	10 (6·6)
Other	8 (5·3)	5 (3·3)
Number of severe exacerbations in previous 12 months		
0	14 (9·3)	11 (7·2)
1	86 (57·0)	75 (49·3)
2	29 (19·2)	31 (20·4)
3	10 (6·6)	22 (14·5)
4	5 (3·3)	6 (3·9)
≥5	7 (4·6)	7 (4·6)
LABA use	92 (60·9)	103 (67·8)
Combination ICS plus LABA inhaler	73 (48%)	82 (54%)
Use of written asthma self-management plan	15 (10%)	20 (13%)
Current smokers	30 (19·9)	29 (19·1)
Ex-smokers	49 (32·5)	48 (31·6)
Non-smokers	72 (47·7)	75 (49·3)

*ACQ* asthma control questionnaire, *FEV*_*1*_ forced expiratory volume in one second, *ICS* inhaled corticosteroids, *LABA* long acting beta-agonist

^a^ As per the ACQ question 6. ACQ question 6 is a categorical score of beta-agonist use over the preceding 7 days in the following bands: score 0—none; score 1—1 to 2 salbutamol inhalations most days; score 2—3 to 4 salbutamol inhalations most days; score 3—5 to 8 salbutamol inhalations most days; score 4—9 to 12 salbutamol inhalations most days; score 5—13 to 16 salbutamol inhalations most days; score 6—more than 16 salbutamol inhalations most days.

### High, marked and extreme beta-agonist use episodes without medical review within 48 h

The proportions of participants with at least one high, marked and extreme use episode were similar in each regimen (Tables 2a–c, respectively). The total number of days of high, marked and extreme use were greater in the Standard regimen (Tables 2a –c, respectively).

**Table 2a:** High beta-agonist use without medical review

Outcome	SMART group (*N* = 151)				Standard group (*N* = 152)				Relative risk or rate SMART vs Standard (95% CI)		*P*
	*N* (%)	Mean (SD)	Median (IQR)	Range	*N* (%)	Mean (SD)	Median (IQR)	Range	Risk	Rate	
At least one episode of high use	84 (56)				68 (45)				1·24 (0·99–1·56)	–	0·058
Number of days of high use		5·1 (14·3)	1 (0–3)	0–130		8·9 (20·9)	0 (0–5)	0–149	–	0·58 (0·39–0·88)	0·010
Number of days of high use in participants with at least one high use episode		9·1 (18·2)	2 (1–7·5)	1–130		19·9 (27·7)	7 (2–26·5)	1–149	–	–	–
Number of days of high use without medical review within 48 h in participants with at least one high use episode		8·5 (17·8)	2 (1–6·5)	0–130		18·3 (24·8)	5 (2–26)	1–123	–	0·49 (0·31–0·75)	0·001
Proportion of high overuse days without medical review within 48 h compared to total high overuse days in participants with at least one high use episode		0·94 (0·20)	1 (1–1)	0–1		0·94 (0·15)	1 (1–1)	0·25 –1	–	–	–

**Table 2b:** Marked beta-agonist overuse without medical review

Outcome	SMART group (*N* = 151)				Standard group (*N* = 152)				Relative risk or rate SMART vs Standard (95% CI)		*P*
	*N* (%)	Mean (SD)	Median (IQR)	Range	N (%)	Mean (SD)	Median (IQR)	Range	Risk	Rate	
At least one episode of marked overuse	54 (36)				56 (37)				0·97 (0·72–1·31)	–	0·85
Number of days of marked overuse		2·6 (10·2)	0 (0–1)	0–109		4·8 (14·9)	0 (0–2)	0 to 144	–	0·56 (0·35–0·88)	0·013
Number of days of marked overuse in participants with at least one marked overuse episode		7·4 (16·0)	2 (1–7)	1–109		13·1 (22·3)	4·5 (2 to 15)	1–144	–	–	–
Number of days of marked overuse without medical review within 48 h in participants with at least one marked overuse episode		6·7 (15·7)	2 (1–6)	1–109		11·7 (19·0)	4 (2–14)	0–118	–	0·62 (0·37–1·06)	0·079
Proportion of marked overuse days without medical review within 48 h compared to total marked overuse days in participants with at least one high use episode		0·94 (0·17)	1 (0·74–1)	0·15–1		0·92 (0·19)	1 (0·96–1)	0–1	–	–	–

**Table 2c:** Extreme beta-agonist overuse without medical review

Outcome		SMART group (*N* = 151)				Standard group (*N* = 152)			Relative risk or rate SMART vs Standard (95% CI)		*P*
	N (%)	Mean (SD)	Median (IQR)	Range	N (%)	Mean (SD)	Median (IQR)	Range	Risk	Rate	
At least one episode of extreme overuse	41 (27)				40 (26)				1·03 (0·71–1·50)	–	0·87
Number of days of extreme overuse		1·6 (6·7)	0 (0–1)	0–75		2·9 (12·2)	0 (0–1)	0–137	–	0·56 (0·34–0·91)	0·02
Number of days of extreme overuse in participants with at least one extreme overuse episode		5·8 (11·9)	2 (1–6)	1–75		11·0 (22·1)	4·5 (2·5–11)	1–137	–	–	–
Number of days of extreme overuse without medical review within 48 h in participants with at least one extreme overuse episode		5·2 (11·9)	2 (1–4)	0–75		9·6 (18·3)	4·5 (2–10)	1–112	–	0·59 (0·31–1·10)	0·096
Proportion of extreme overuse days without medical review within 48 h compared to total extreme overuse days in participants with at least one high use episode		0·90 (0·26)	1 (1–1)	0–1		0·94 (0·15)	1 (1–1)	0·29–1	–	–	–

**Fig. 1:**
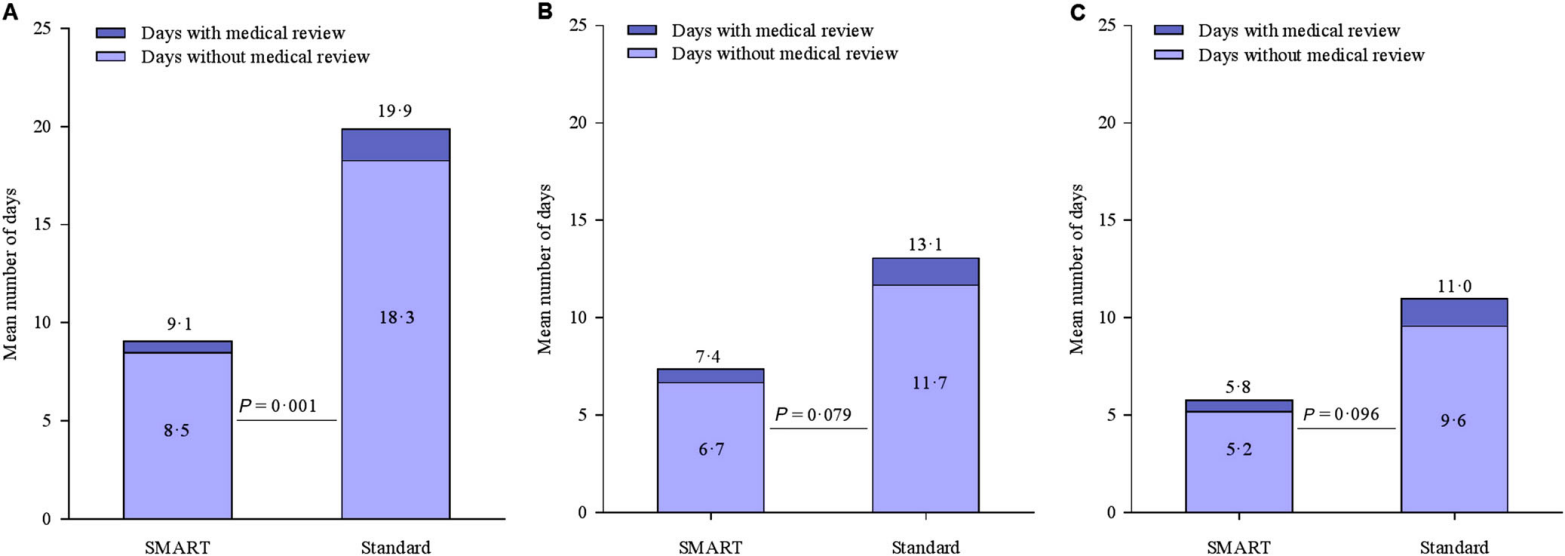
**a** Mean number of high use days in participants with at least one high use episode, and mean number of high use days without medical review within 48 h in participants with at least one high use episode. **b** Mean number of marked overuse days in participants with at least one marked overuse episode, and mean number of marked overuse days without medical review within 48 h in participants with at least one marked overuse episode. **c** Mean number of extreme overuse days in participants with at least one extreme overuse episode, and mean number of extreme overuse days without medical review within 48 h in participants with at least one extreme overuse episode

In the SMART regimen, the number of high use days without medical review within 48 h was approximately half of that in the Standard regimen; relative rate (95% CI) 0·49 (0·31 to 0·75), *P* = 0·001 (Fig. 1, Table 2a). The point estimate of association was consistent with a lower number of marked and extreme use days without medical review within 48 h with the SMART regimen (Fig. 1 and Tables 2b, 2c, respectively).

In both the SMART and Standard regimens, the proportion of high, marked and extreme use days without medical review within 48 h, compared to total days of high, marked and extreme use was at least 90% (Tables 2a, 2b and 2c, respectively).

### Severe exacerbations of asthma

During the study period, at least one severe asthma exacerbation was experienced by 28/151 (18·5%) of SMART participants and 50/152 (32·9%) of Standard participants. There was a total of 35 and 66 severe exacerbations in the SMART and Standard groups respectively; relative rate (95% CI) 0·54 (0·36 to 0·82), *P* = 0·004.

### Daily medication use prior to and following severe exacerbations

**Fig. 2:**
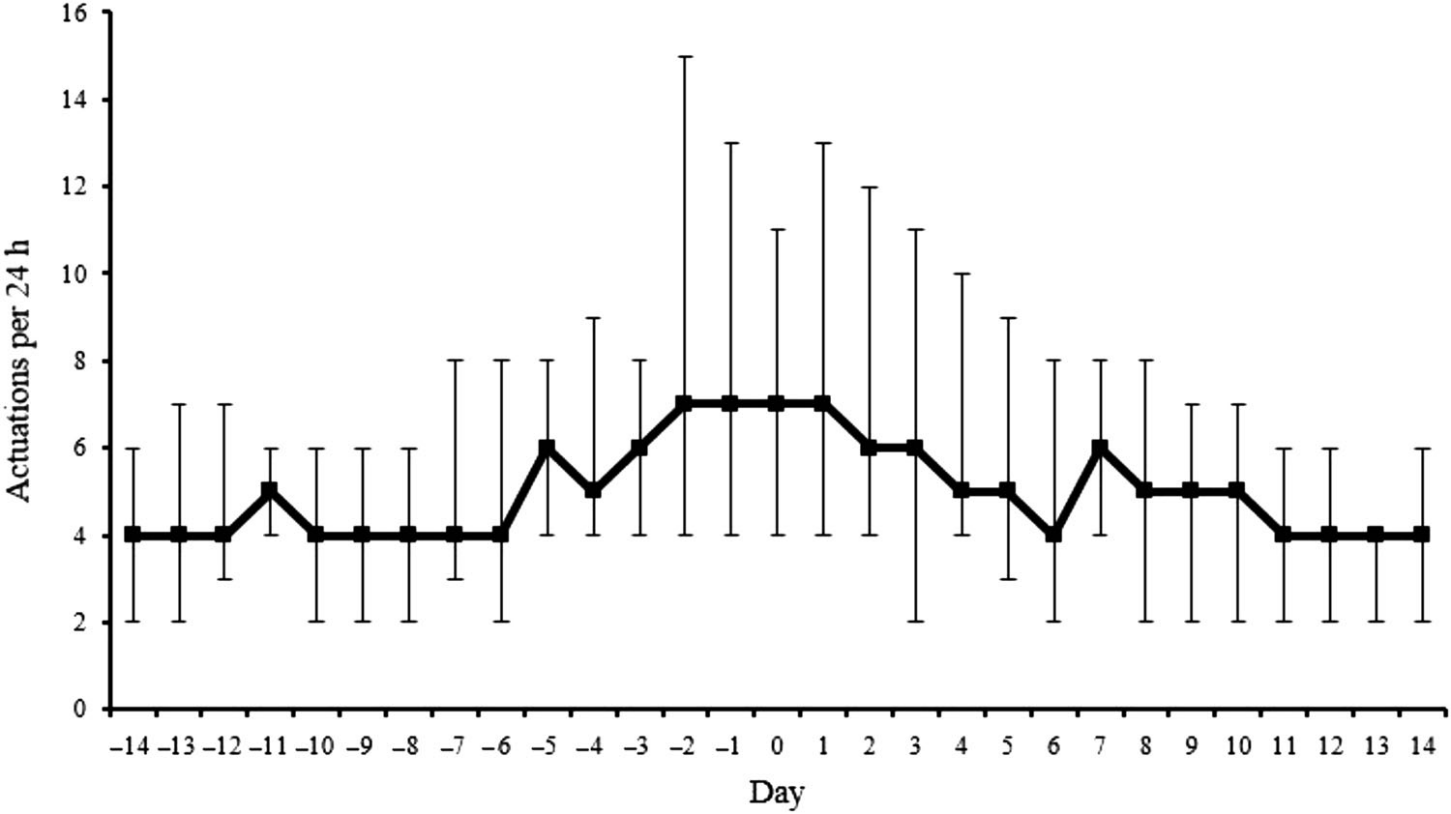
Median [interquartile range (IQR)] daily budesonide/formoterol actuations in the 14 days prior to, and the 14 days following a severe exacerbation in the SMART group. Day 0 represents the initiation of corticosteroid therapy. There were 35 severe exacerbations in the SMART group. For patients with repeat exacerbations, a minimal interval of 28 days between exacerbations was required, to avoid overlap of data within the same participant. One patient in the SMART group had a second severe exacerbation within 28 days following the prior exacerbation. Thus, electronic data for 34 severe exacerbations were included in the plots of medication use patterns for the SMART group

**Fig. 3:**
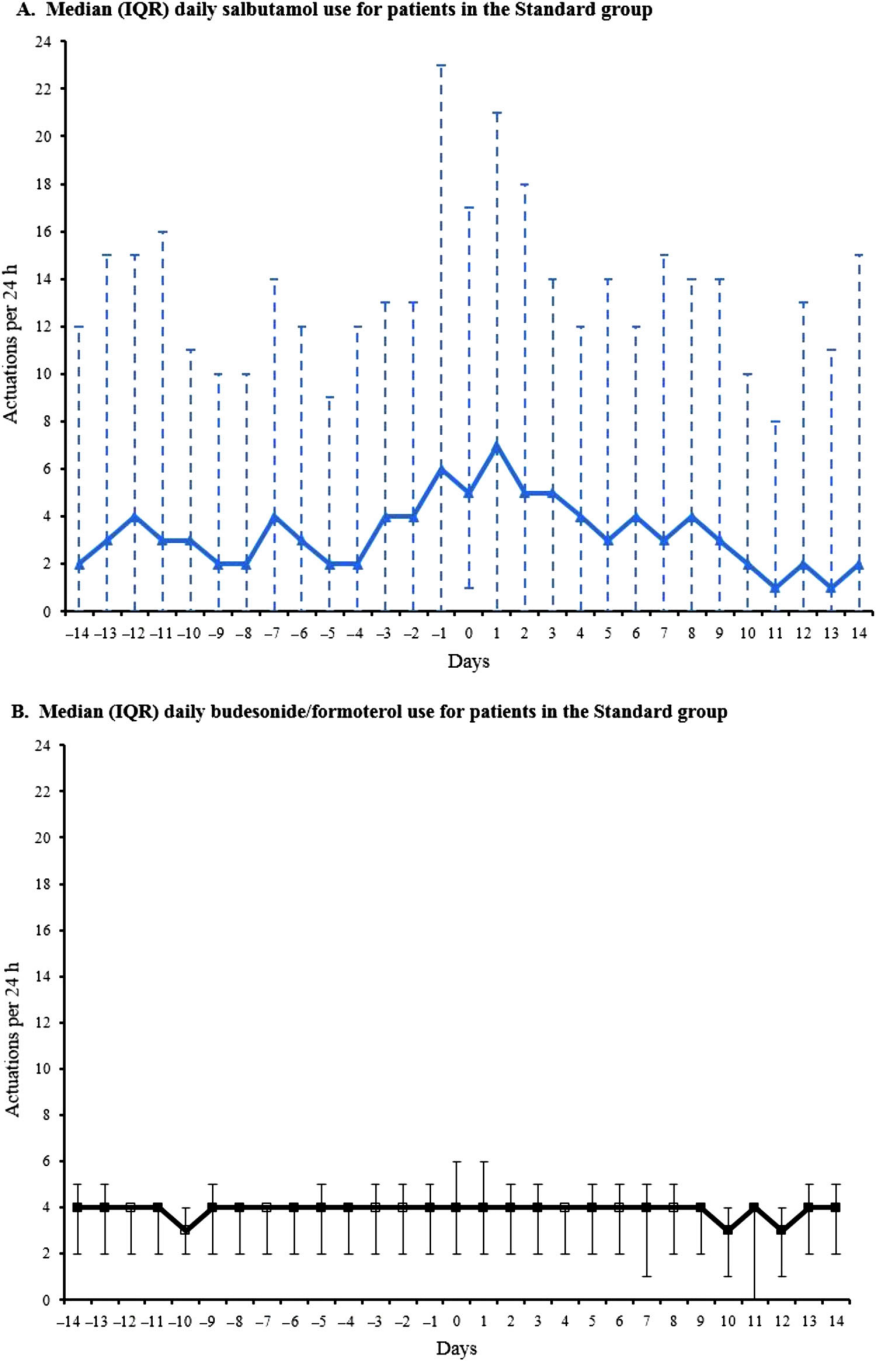
Median (IQR) daily salbutamol **a** and budesonide/formoterol **b** actuations in the 14 days prior to, and the 14 days following a severe exacerbation in the Standard group. Day 0 represents the initiation of corticosteroid therapy. There were 66 severe exacerbations in the Standard group. For patients with repeat exacerbations, a minimal interval of 28 days between exacerbations was required, to avoid overlap of data within the same participant. Three patients in the Standard group had repeat severe exacerbations occurring within 28 days following the prior exacerbation. In another patient randomised to Standard treatment, there was no recorded data for two severe exacerbations due to use of non-study inhaled medication. Thus, electronic data for 61 severe exacerbations were included in the plots of medication use patterns in the Standard group. For these plots, only one of the included days (2 days prior to severe exacerbation for a patient in the Standard group) met dose dumping criteria (≥100 actuations in a 3 h period)

Medication use in the 14 days prior to and 14 days following a severe exacerbation for the SMART and Standard regimens is shown in Figs. 2 and 3, respectively. With the SMART regimen, there was increasing budesonide/formoterol use from about 5 days prior to initiation of oral corticosteroid therapy, with high use for a further 5 days before returning to baseline levels after 10 days. With the Standard regimen, there was variable salbutamol use from about 14 days prior to initiation of oral corticosteroid therapy, increasing markedly the day before, with high use for a further 3 days before returning to baseline levels after 10 days. In the severe exacerbation dataset, only one of the included days, 2 days prior to severe exacerbation for a patient in the Standard group, met dose dumping criteria, defined as greater than or equal to 100 actuations in a 3-h period.^18^

The number of days in which high, marked or extreme use occurred in the 14 days prior to a severe exacerbation, expressed as a percentage of all high, marked and extreme use days was 56/766 (7%), 28/400 (7%) and 10/237 (4%) for SMART, and 166/1355 (12%), 111/735 (15%) and 80/438 (18%) for Standard, respectively.

Of the high use episodes in the 14-day period prior to a severe exacerbation, 38/56 (68%) and 133/166 (80%) were not associated with medical review within 48 h, in the SMART and Standard regimens, respectively. Of the marked overuse episodes in the 14-day period prior to a severe exacerbation, 18/28 (64%) and 87/111 (78%) were not associated with medical review within 48 h, in the SMART and Standard regimens, respectively. Finally, of the extreme overuse episodes in the 14-day period prior to a severe exacerbation, 5/10 (50%) and 65/80 (81%) were not associated with medical review within 48 h, in the SMART and Standard regimens, respectively.

### Highest medication use prior to and following severe exacerbations

In the SMART group, the median (range) highest daily number of budesonide/formoterol actuations was 11 (4 to 63) over the 14 days before severe exacerbations. In the Standard group, the median (range) highest daily number of salbutamol actuations was 13 (0 to 150) over the 14 days before severe exacerbations; on the day of highest salbutamol use preceding severe exacerbations, the median (range) number of budesonide/formoterol actuations was 4 (0 to 84). On this day of maximal salbutamol use, there were 8/61 (13%) instances when >8 actuations of budesonide/formoterol were taken and 10/61 (16%) instances when zero actuations of budesonide/formoterol were taken.

In the SMART group, the median (range) highest daily number of budesonide/formoterol actuations was 10 (0 to 71) in the 14 days after oral corticosteroid use for severe exacerbations (Day 0). In the Standard group, the median (range) highest daily number of salbutamol actuations was 20 (0 to 152) in the 14 days after Day 0; on the day of highest salbutamol use following Day 0, the median (range) number of budesonide/formoterol actuations was 4 (0 to 33). On this day of maximal salbutamol use, there were 7/61 (11%) instances when >8 actuations of budesonide/formoterol were taken and 9/61 (15%) instances when zero actuations of budesonide/formoterol were taken.

## Discussion

This study shows that in a high-risk population of adult patients with asthma, around 90% of beta-agonist overuse episodes, well in excess of the asthma action plan thresholds recommending prompt medical review, do not lead to patients obtaining medical review within 48 h. It is likely that the symptomatic relief obtained from the high doses of beta-agonist may be one of the underlying causes for the delay in or lack of seeking medical help in the situation of such poorly controlled, worsening asthma.

We observed that in around 90% of marked and extreme overuse episodes, patients did not obtain medical review within 48 h despite this use being at least 1·5 and 2-fold higher than the level of use at which their asthma action plan advised that medical care must be sought. These findings complement our previous observation that 93% of high-use episodes, in which patients took at least 16 actuations of salbutamol or equivalent in a 24-h period, occurred without medical review within the next 48 h, despite this advice being documented in the provided asthma action plans.^16^ These findings were unexpected as they suggest that increasing beta-agonist use from at least 16 to at least 32 actuations of salbutamol or equivalent in a 24-h period did not lead to a greater likelihood of a patient seeking medical care.

Patterns of beta-agonist use were also examined in the patients in whom a severe exacerbation was confirmed with the prescription of systemic corticosteroids. In the SMART regimen, the mean daily use of budesonide/formoterol progressively increased 5 days before the index corticosteroid use and remained high for a further 5-day period before returning close to baseline levels after 10 days. For the Standard regimen, the salbutamol use was variable from about 14 days prior to the index course of corticosteroids, increasing markedly the day before, with the high level of use gradually returning to baseline levels over 10 days. The majority of overuse episodes in the 14-day period prior to the initiation of oral corticosteroid therapy were not associated with medical review within 48 h, regardless of the magnitude of the overuse. These findings suggest that even in patients who did seek medical review, there is often delay during worsening asthma, resulting in the later prescription of oral corticosteroids in a severe exacerbation. This may have contributed to our finding that in the setting of a severe exacerbation it takes at least 5 days of oral corticosteroid treatment before there is sufficient improvement to result in a reduction in beta-agonist use, regardless of whether the SMART or Standard regimen was used.

Different patterns of budesonide/formoterol use were observed in the Standard group during severe exacerbations. Some patients used no budesonide/formoterol on the day of highest salbutamol use preceding the severe exacerbation. This non-adherence may contribute to progressive worsening of unstable asthma, increasing the risk of a severe exacerbation. Another pattern was the use of budesonide/formoterol as an as required ‘reliever’ medication well in excess of the maintenance four actuations per day. These observations suggest that some patients may use their maintenance budesonide/ formoterol inhaler according to presumed need, similar to the SMART regimen, despite being prescribed fixed dose maintenance treatment and salbutamol for reliever.

Another important observation was that while the SMART regimen reduced the number of high, marked and extreme overuse episodes by at least 40%, it did not influence the likelihood of a patient obtaining medical review when an overuse episode occurred. As a result, the SMART regimen reduced the total number of overuse episodes in which patients did not obtain medical review within 48 h, to a similar extent to which it reduced the total number of overuse episodes. Together with the 46% reduction in severe exacerbations, these findings suggest that the SMART regimen has a better safety profile as it reduced the total number of overuse episodes in which delay in obtaining medical review occurred.

There are a number of methodological issues that require consideration. As an open-label study, there was the potential for bias, however the use of a single inhaler in the SMART regimen and separate inhalers in the Standard regimen without the requirement for dummy placebo inhalers enhanced generalisability of the findings to real world clinical practice. Metered dose inhaler (MDI)’s were used rather than dry powder inhalers (Turbuhaler) to deliver budesonide/formoterol, as it enabled us to use validated electronic monitors of inhaler use.^19, 20^ Beta-agonist overuse related to the measures of budesonide/formoterol and salbutamol for the SMART and Standard regimens, respectively. This is likely to have led to an underestimation of the number of beta-agonist overuse episodes for the Standard regimen, as it did not take into account the use of budesonide/formoterol as a reliever additional to the four maintenance actuations per day, a behaviour which was noted in some patients.^17^

There are several additional clinical implications of these findings. Firstly, the patient’s failure to recognise that the need for frequent and high doses of beta-agonist indicates severe asthma, likely contributes to the delay in or lack of seeking medical help. Secondly, this behaviour may reflect previous experience of these high risk patients with asthma, that most exacerbations of asthma requiring high doses of beta-agonist are self-limiting and eventually resolve without medical review, oral corticosteroids, or hospital attendance. This interpretation is supported by our observation that over 80% of all days of beta-agonist overuse, regardless of extent, did not lead to a course of oral steroids within the following 14 days. The higher proportions associated with the SMART regimen suggest that budesonide/formoterol overuse may be more likely to lead to resolution of the severe asthma episode without oral steroids than salbutamol overuse. Thirdly, even in the setting of an RCT with repeat clinic visits during which the asthma action plan was introduced and reinforced, most patients did not follow this written advice to obtain medical review when beta agonist use exceeded predefined levels of increased use.

In conclusion, in at least 90% of days in which beta-agonist overuse occurred, patients with high-risk asthma did not obtain medical review within 48 h, regardless of the magnitude of the beta-agonist overuse, or the inhaled corticosteroid/long-acting beta-agonist regimen.

## Methods

### Design

This is a secondary analysis of data from a 24-week multicentre, open-label RCT of Single combination budesonide/formoterol inhaler as Maintenance And Reliever Therapy (SMART regimen) vs. fixed-dose budesonide/formoterol with salbutamol for relief (Standard regimen) in 303 adult patients with a physician’s diagnosis of asthma, described previously.^16^ Patients were eligible if they had a current prescription for inhaled corticosteroids (ICS) and at least one asthma exacerbation in the preceding year. An exacerbation was defined as presentation to a General Practice or Emergency Department resulting in prescription of oral corticosteroids or treatment with spacer-delivered or nebulised bronchodilator, or self-administration of prednisone for asthma for at least 3 days. Exclusion criteria included a diagnosis of chronic obstructive pulmonary disease and current or ex-smokers with a > 10 pack-year smoking history with onset of respiratory symptoms after the age of 40.

Patients were randomised to the SMART regimen: a MDI containing 200/6 µg budesonide/formoterol (Vannair, AstraZeneca Limited, Auckland, New Zealand, which is the MDI formulation of Symbicort turbuhaler), two actuations twice daily for maintenance with one extra dose as needed for relief; or the Standard regimen: 200/6 µg Vannair MDI, two actuations twice daily for maintenance with 100 µg salbutamol MDI (Ventolin, GlaxoSmithKline Limited, Auckland, New Zealand), one to two extra actuations for relief. All participants were given written asthma action plans and verbal instructions outlining when to consult their doctor. All participants provided written informed consent and the trial was approved by the New Zealand Multi Region Ethics Committee, and prospectively registered [ACTRN 12610000515099]. The study protocol is available at http://www.mrinz.ac.nz/uploads/mrinz/SMART_Protocol.pdf

All Vannair and Ventolin MDIs incorporated an electronic monitor (Smartinhaler Tracker, Nexus6 Limited, Auckland, New Zealand), which recorded the date and time (to the nearest second) each time an inhaler was actuated. These monitors were 99·7% accurate in recording actuations in bench testing^19^ and were used in accordance with strict trial quality control processes.^20^ Participants were not aware of the exact capabilities of the monitors.

### Definitions of high, marked and extreme beta-agonist use episodes

#### High use

For SMART, this was defined as >8 actuations of budesonide/formoterol in excess of the four maintenance doses per 24-h period i.e., equivalent to >12 actuations in total. For Standard, this was defined as >16 actuations of salbutamol per 24-h period. These thresholds were based on the dose limits of beta-agonist use requiring medical review, defined by the action plans implemented in this study,^21, 22^ and supported by the results of the short-term bronchodilator equivalence of formoterol 6 µg to salbutamol 200 µg, with repeat dosing in acute asthma.^23, 24^ In accordance with their action plans, participants were advised to seek medical review at these high use thresholds.

#### Marked use

For SMART, this was defined as >12 actuations of budesonide/formoterol in excess of the four maintenance doses per 24-h period i.e., equivalent to >16 actuations in total. For Standard, this was defined as >24 actuations of salbutamol per 24-h period. These thresholds were based on 1.5 times the limits of beta-agonist use requiring medical review, defined by the action plans.^21, 22^

#### Extreme use

For SMART, this was defined as >16 actuations of budesonide/formoterol in excess of the four maintenance doses per 24-h period i.e., equivalent to >20 actuations in total. For Standard, this was defined as >32 actuations of salbutamol per 24-h period. These thresholds were based on twice the limits of beta-agonist use requiring medical review, defined by the action plans.^21, 22^

### High, marked, and extreme beta-agonist use without medical review

The number of days of high, marked, or extreme beta-agonist use without medical review within 48 h was determined using the following rules: for every high, marked, or extreme beta-agonist use day, the ‘index day’, the database was checked to determine if the patient attended for medical review, which could include primary care clinic, an after-hours clinic or hospital attendance, either on the day of overuse or the next day. This 48-h window was defined as per the Standard action plan, which specifies that the patient should attend for medical review ‘within 1 to 2 days’ in the setting of worsening asthma recognised by symptoms including ‘reliever only lasting 2–3 h’.^22^ The SMART plan advises patients to seek medical review on the same day if more than 12 actuations of budesonide/formoterol are taken.^21^

If the participant attended for medical review, then overuse occurring on the index day, the day of medical review and in the 7 days after medical review was not counted as overuse without medical review, as a ‘stand-down’ period. In effect, overuse occurring on these days was ‘permissible’ as the patient had attended for medical review in the setting of this exacerbation. This criterion was chosen as the american thoracic society (ATS)/european respiratory society (ERS) definition of severe exacerbations separate exacerbations by 7 days.^25^ If a participant attended for repeated medical reviews during the stand-down period, then the 7-day period where overuse without medical review is not counted, was restarted. This approach was used as the object of this analysis was to explore the relationship between the ‘index’ overuse episode and the first episode of medical review following this.

### Outcomes

In this exploratory analysis, no particular primary outcome was specified and there is no adjustment for multiple comparisons. The outcome variables were firstly the proportion of participants in each arm of the RCT with at least one episode of overuse for each type of beta-agonist overuse; high, marked and extreme. A second outcome was the number of days of overuse for each type of beta-agonist overuse. A third outcome was the number of days of overuse without medical review within 48 h for each type of beta-agonist overuse. The final outcome was the proportion of days of overuse without medical review within 48 h compared to the total number of days of overuse for each type of beta-agonist overuse.

Medication use in the 14 days prior to and following a severe exacerbation was also examined. A severe exacerbation was defined according to ATS/ERS criteria^25^ as: (a) the use of systemic corticosteroids for at least 3 days; or (b) a hospitalisation or Emergency Department visit because of asthma, requiring systemic corticosteroids. Courses of corticosteroids separated by 7 days or more were treated as separate severe exacerbations. The date of the first day of corticosteroid therapy was Day 0. Medication use for the 14-day period before and up to 14 days after Day 0 was extracted from the data set for each participant with at least one severe exacerbation. For patients with repeat exacerbations, a minimum interval of 28 days between exacerbations was required to avoid overlap of data within the same participants.

### Statistical methods

The relative risk of at least one episode of overuse by overuse type was by simple contingency table analysis and expressed as a relative risk. The relative rates of days of high, marked and extreme beta-agonist overuse was by Poisson regression with an offset for the adjusted days of treatment exposure, and a further adjustment for over-dispersion. SAS version 9·4 was used.

### Data availability statement

Data is available on application to the corresponding author

## Electronic supplementary material

Supplementary Information

## References

[1] Fraser PM, Speizer FE, Waters SD, Doll R, Mann NM (1971). The circumstances preceding death from asthma in young people in 1968 to 1969. Br. J. Dis. Chest.

[2] Sears MR, Beaglehole R (1987). Asthma morbidity and mortality. J. Allergy Clin. Immunol..

[3] Beasley R, Pearce N, Crane J, Windom H, Burgess C (1991). Asthma mortality and inhaled beta-agonist therapy. Aust. N. Z. J. Med..

[4] Royal College of Physicians. *‘Why asthma still kills’—The National Review of Asthma Deaths (NRAD) Confidential Enquiry report* (Royal College of Physicians, 2014).

[5] Levy ML (2015). The national review of asthma deaths: what did we learn and what needs to change?. Breathe.

[6] Spitzer WO (1992). The use of beta-agonists and the risk of death and near death from asthma. N. Engl. J. Med..

[7] Suissa S (1994). A cohort analysis of excess mortality in asthma and the use of inhaled beta-agonists. Am. J. Respir. Crit. Care Med..

[8] Suissa S, Blais L, Ernst P (1994). Patterns of increasing beta-agonist use and the risk of fatal or near-fatal asthma. Eur Respir J.

[9] de Vries F, Setakis E, Zhang B, van Staa TP (2010). Long-acting beta2-agonists in adult asthma and the pattern of risk of death and severe asthma outcomes: a study with the General Practice Research Database. Eur. Respir. J..

[10] Anderson HR (2005). Bronchodilator treatment and deaths from asthma: case-control study. Br. Med. J..

[11] Windom HH (1990). The self-administration of inhaled beta-agonist drugs during severe asthma. N.Z. Med. J..

[12] Patel M (2013). Accuracy of patient self-report as a measure of inhaled asthma medication use. Respirology.

[13] Rand CS (2012). Mediators of asthma outcomes. J. Allergy Clin. Immunol..

[14] Riekert KA, Rand CS (2002). Electronic monitoring of medication adherence: when is high-tech best?. J. Clin. Psychol. Med. Settings.

[15] Milgrom H (1996). Noncompliance and treatment failure in children with asthma. J. Allergy Clin. Immunol..

[16] Patel M (2013). Efficacy and safety of maintenance and reliever combination budesonide/formoterol therapy in asthma patients at risk of severe exacerbations: a randomised controlled trial. Lancet Respir. Med..

[17] Patel M (2015). The use of β_2_-agonist therapy before hospital attendance for severe exacerbations: a post-hoc analysis. Prim. Care Respir. J..

[18] Rand CS (1992). Metered-dose inhaler adherence in a clinical trial. Am. Rev. Respir. Dis..

[19] Patel M (2012). Six-month in vitro validation of a metered dose inhaler electronic monitoring device: implications for asthma clinical trial use. J. Allergy Clin. Immunol..

[20] Patel M (2013). Use of metered-dose inhaler electronic monitoring in a real-world asthma randomized controlled trial. J. Allergy Clin. Immunol Pract..

[21] National Asthma Council. *SMART Asthma Action Plan* https://s3-ap-southeast-2.amazonaws.com/assets.nationalasthma.org.au/resources/341-SMART-Asthma-Action-Plan-Rapihaler-only-English.pdf (2013).

[22] Holt S, Masoli M, Beasley R (2004). The use of the self-management plan system of care in adult asthma. Prim. Care Respir. J..

[23] Balanag VM, Yunus F, Yang PC, Jorup C (2006). Efficacy and safety of budesonide/formoterol compared with salbutamol in the treatment of acute asthma. Pulm. Pharmacol. Ther..

[24] Rubinfeld AR (2006). Formoterol Turbuhaler as reliever medication in patients with acute asthma. Eur. Respir. J..

[25] Reddel HK, Taylor DR, Bateman ED (2009). An official American Thoracic Society/European Respiratory Society statement: asthma control and exacerbations: standardizing endpoints for clinical asthma trials and clinical practice. Am. J. Respir. Crit. Care Med..

