# Supplementary material for: Beta-agonist overuse and delay in obtaining medical review in high risk asthma: a secondary analysis of data from a randomised controlled trial
Source: NPJ Prim Care Respir Med. 2017 May 11;27:33. doi: 10.1038/s41533-017-0032-z (PMC5435086; doi:10.1038/s41533-017-0032-z)
Supplement: Supplementary file 1 — Supplementary Information [file 41533_2017_32_MOESM1_ESM.pdf]

The SMART Study Group

Steering committee: Mitesh Patel (clinical co-ordinating investigator), Janine Pilcher, Alison Pritchard, Kyle Perrin, Justin Travers, Dominick Shaw, Shaun Holt, Matire Harwood, Peter Black, Mark Weatherall (study biostatistician), Richard Beasley (principal investigator); Auckland (Henderson Medical Centre): Clare McGuinness-Goodwin, Bill Mackey, Rodney Marks, Vikky Qi, Tyronne Tranquilino, Dirk Venter;

Auckland (University of Auckland): Amy Chan;
Lower Hutt (Tu Kotahi Maori Asthma Trust): Cheryl Davies, Ann Smith;
Sydney (Woolcock Institute of Medical Research): Helen K Reddel;
Tauranga (CentralMed General Practice): Andrew Corin, Colin Helm, Chris Tofield;
Tauranga (Papamoa Pines Medical Centre): Davitt Sheahan;
Wellington (BoydHQ Limited): Craig Boyd (database engineer);
Wellington (MRINZ): Tanya Baker, Denise Fabian, Alexander Hosking, Claire Munro, Maureen Stretch, Mathew Williams.
